# Supplementary material for: Ecological dependencies make remote reef fish communities most vulnerable to coral loss
Source: Nat Commun. 2021 Dec 14;12:7282. doi: 10.1038/s41467-021-27440-z (PMC8671472; doi:10.1038/s41467-021-27440-z)
Supplement: Supplementary file 1 — Supplementary Information File [file 41467_2021_27440_MOESM1_ESM.pdf]

Supplementary Figures

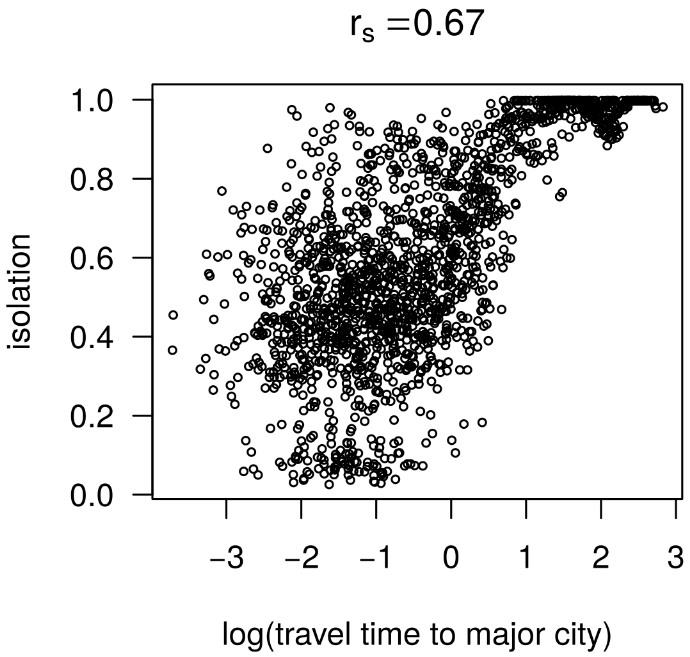

Supplementary Figure 1 | Reef remoteness correlates with isolation. Remoteness was measured as travel time in $\log_e$ transformed hours from the target reef locality to the closest major city[21,], while isolation was measured, for each 1° × 1° reef locality, as 1 minus the fraction of land within 5° latitude/longitude from the target locality[27]**.** Correlation strength was assessed as Spearman's rank correlation coefficient (r_s).

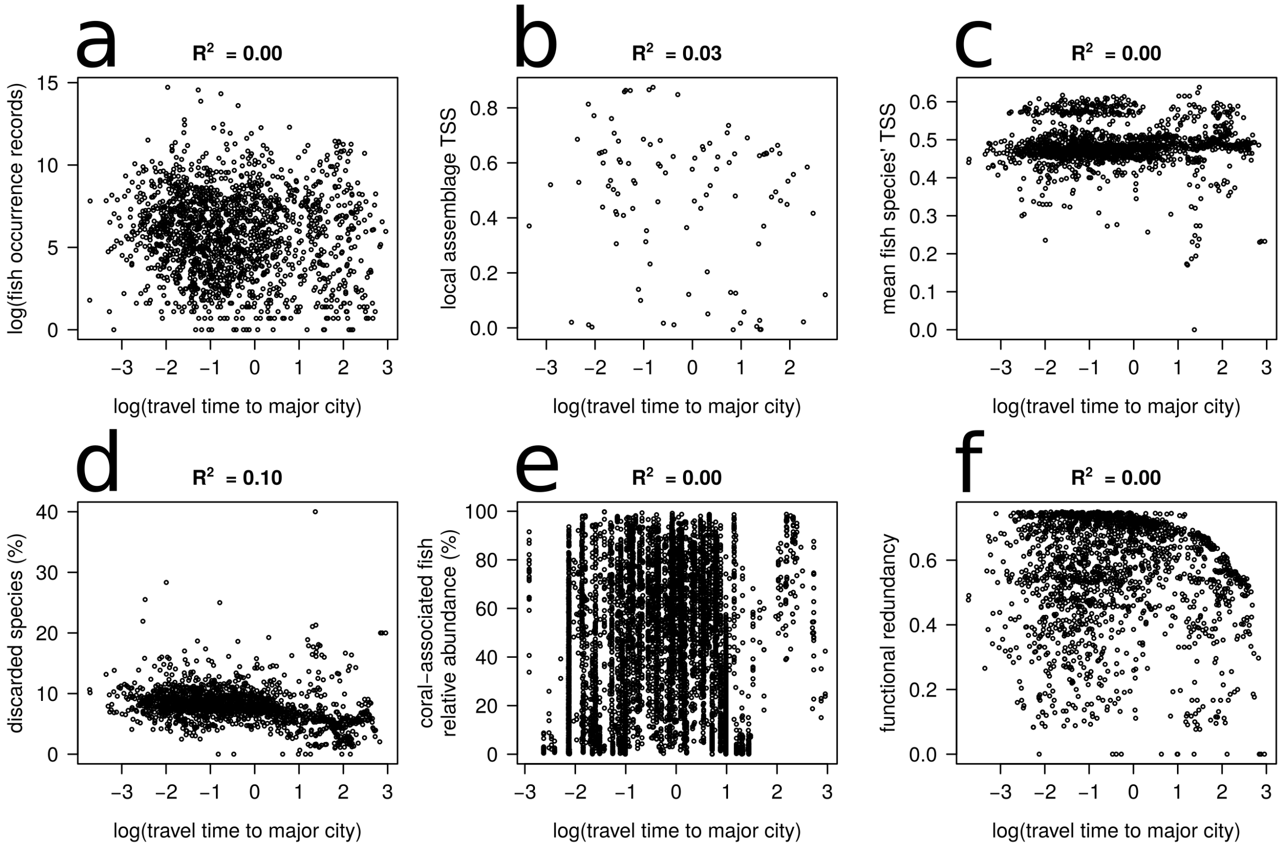

Supplementary Figure 2 | The negative relationship between distance from human settlements and fish-coral dependency is not biased by variation in data availability across remoteness values. Plots reports comparisons across 1° × 1° reef localities between remoteness (measured as travel time to the closest major city, in log_e transformed hours[21]) and: (**a**) number of fish records available from OBIS[36] and GBIF[37]; (**b**) TSS values obtained from the comparison between the species ranges devised with our procedure and independent species distribution data from the GASPAR[40] dataset (in all 1° × 1° reef localities covered by the latter, n=102); (**c**) average of individual species' TSS values obtained by comparing the distribution of a target species devised by our procedure with that according to the GASPAR dataset; (**d**) fraction of fish species to be discarded in each locality due to lack of ecological information; (**e**) relative abundance (%) of coral-associated species, assessed from the Reef Life Survey dataset[52]; (**f**) fish functional redundancy measured as 1 minus the ratio between the number of unique functional entities (identified as in Mouillot et al.[53]) and total species richness.

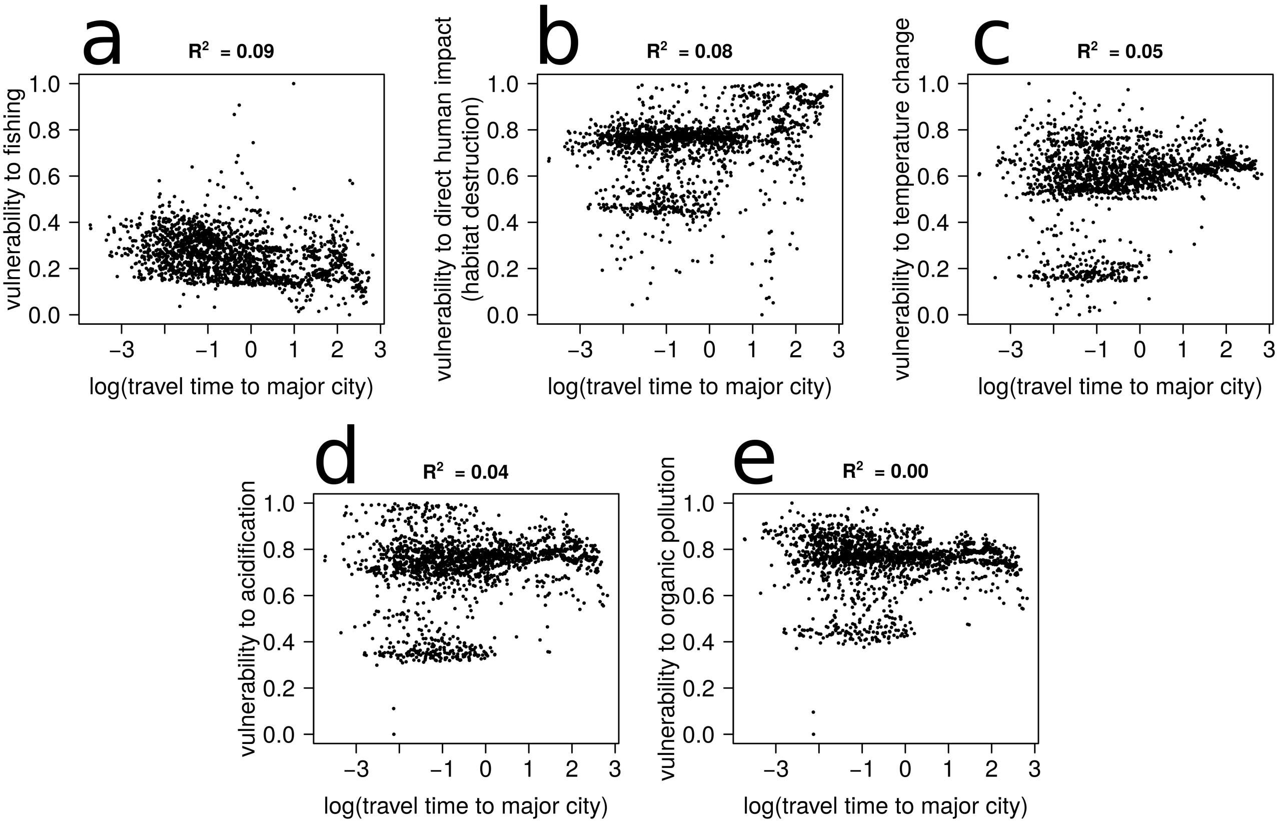

Supplementary Figure 3 | Quantitative support to the assumption of no relationships between local and global hazard specific vulnerabilities and remoteness. a, Comparison between remoteness (quantified as travel time in $\log_e$ transformed hours from the target reef locality to the closest major city[21]) and average vulnerability to fishing of all fish species in each reef locality, assessed using the vulnerability to fishing measure provided by FishBase[38,51]; **b**, comparison between remoteness and fish community vulnerability to direct human impacts (habitat destruction) based on species habitat preference as defined by FishBase[38], and computed as the fraction of demersal, benthopelagic and coral associated species per reef locality; **c-e**, comparison between remoteness and proxies of community tolerance to changes in environmental conditions, computed as the average of species environmental tolerance ranges per locality.

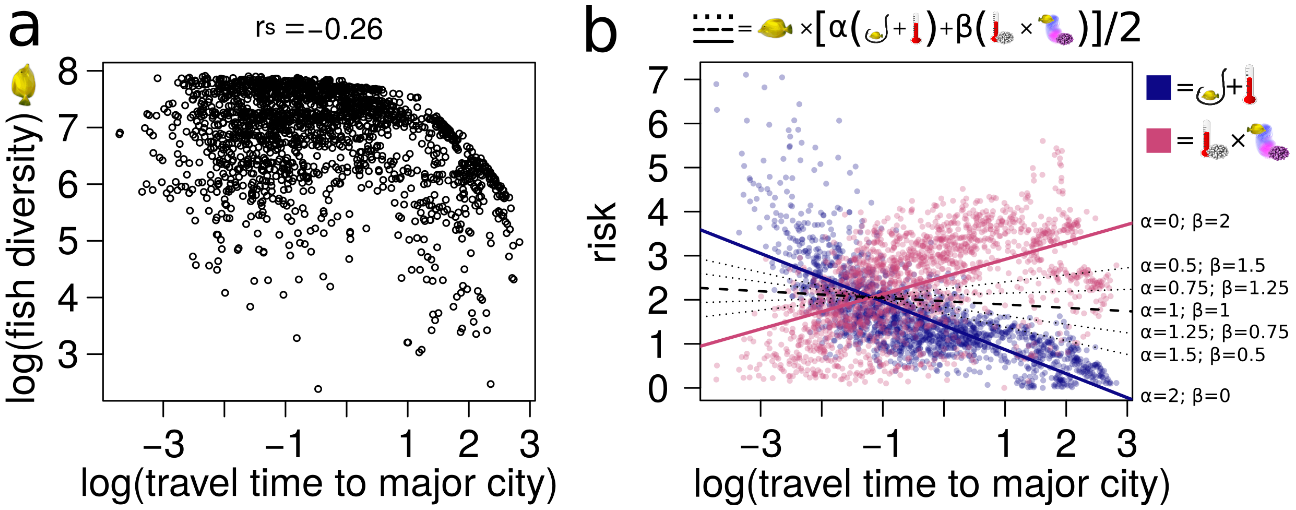

Supplementary Figure 4 | How fish-coral dependency modifies the risk-remoteness relationship when exposure is taken into account. Coral reef remoteness was quantified as travel time (in $\log_e$ transformed hours) from the target reef locality to the closest major city[21]. Exposure was quantified as $\log_e$ transformed local fish diversity. The blue dots represent risk quantified as the sum of threats from local + global hazards on reefs (as in Fig. 2d), while magenta dots represent risk quantified as bleaching susceptibility × fish-coral dependency. Both components of risk (i.e. local+global hazards and bleaching susceptibility × fish-coral dependency) were rescaled between 0 and 1. The two rescaled risks component are then combined into a single risk assessment equation where risk = log(fish diversity) × [α (local+global hazards) + β (bleaching susceptibility × fish-coral dependency)]/2. The lines in the plot represent the slopes of the trend lines from different parametrizations of the risk equation. When equal weight is given to the two risk components, risk remains almost perfectly constant across remoteness values (trend line slope = -0.08, black dashed line).
