# Supplementary material for: Ecological dependencies make remote reef fish communities most vulnerable to coral loss
Source: Nat Commun. 2021 Dec 14;12:7282. doi: 10.1038/s41467-021-27440-z (PMC8671472; doi:10.1038/s41467-021-27440-z)
Supplement: Supplementary file 3 — Reporting summary [file 41467_2021_27440_MOESM3_ESM.pdf]

nature research

Corresponding author(s): Giovanni Strona

Last updated by author(s): Nov 4, 2021

Reporting Summary

Nature Research wishes to improve the reproducibility of the work that we publish. This form provides structure for consistency and transparency in reporting. For further information on Nature Research policies, see our Editorial Policies and the Editorial Policy Checklist.

Statistics

For all statistical analyses, confirm that the following items are present in the figure legend, table legend, main text, or Methods section.

n/a	Confirmed	
☐	☒	The exact sample size (n) for each experimental group/condition, given as a discrete number and unit of measurement
☒	☐	A statement on whether measurements were taken from distinct samples or whether the same sample was measured repeatedly
☐	☒	The statistical test(s) used AND whether they are one- or two-sided<br>*Only common tests should be described solely by name; describe more complex techniques in the Methods section.*
☒	☐	A description of all covariates tested
☒	☐	A description of any assumptions or corrections, such as tests of normality and adjustment for multiple comparisons
☐	☒	A full description of the statistical parameters including central tendency (e.g. means) or other basic estimates (e.g. regression coefficient) AND variation (e.g. standard deviation) or associated estimates of uncertainty (e.g. confidence intervals)
☐	☒	For null hypothesis testing, the test statistic (e.g. F, t, r) with confidence intervals, effect sizes, degrees of freedom and P value noted<br>*Give P values as exact values whenever suitable.*
☒	☐	For Bayesian analysis, information on the choice of priors and Markov chain Monte Carlo settings
☒	☐	For hierarchical and complex designs, identification of the appropriate level for tests and full reporting of outcomes
☐	☒	Estimates of effect sizes (e.g. Cohen's d, Pearson's r), indicating how they were calculated

Our web collection on statistics for biologists contains articles on many of the points above.

Software and code

Policy information about availability of computer code

Data collection: We collected data using custom scripts in Python2.7 and Python3 whenever possible, we downloaded them manually otherwise.

Data analysis: We analyzed data using custom code written in Python. All the data and scripts permitting to replicate the analyses are available from https://github.com/giovannistrona/risk_remoteness. To compute reef remoteness, we used the free software SAGA GIS, System for automated geoscientific analyses (2013); www.saga-gis.org/en/index.html. To perform machine learning analysis we used Scikit-learn version 0.23.1.

For manuscripts utilizing custom algorithms or software that are central to the research but not yet described in published literature, software must be made available to editors and reviewers. We strongly encourage code deposition in a community repository (e.g. GitHub). See the Nature Research guidelines for submitting code & software for further information.

Data

Policy information about availability of data

All manuscripts must include a data availability statement. This statement should provide the following information, where applicable:

- Accession codes, unique identifiers, or web links for publicly available datasets
- A list of figures that have associated raw data
- A description of any restrictions on data availability

All the data used in the analysis are freely available online from:

1. reef distribution map: http://data.unep-wcmc.org/datasets/1;
2. fish occurrence data: https://obis.org/; and GBIF (Actinopterygii, https://doi.org/10.15468/dl.k6vam4; Elasmobranchii, https://doi.org/10.15468/dl.pu4tcx; Holocephali, https://doi.org/10.15468/dl.npckhm; Sarcopterygii, https://doi.org/10.15468/dl.huzujv);
3. fish ecology data: http://www.fishbase.org;
4. ocean impact layers: https://knb.ecoinformatics.org/view/doi:10.5063/F12B8WBS;

5. the friction surface map needed to compute accessibility: https://malariaatlas.org/research-project/accessibility-to-cities;
6. human settlement data: http://data.europa.eu/89h/jrc-ghsl-ghs_smod_pop_globe_r2016a;
7. bleaching alert data: https://coralreefwatch.noaa.gov;
8. environmental layers: https://www.bio-oracle.org;
9. marine eco-regions: https://data.unep-wcmc.org/datasets/38;
10. fish trophic interactions: https://www.globalbioticinteractions.org;
11. reef fish abundance data: https://portal.aodn.org.au/search.
12. GASPAR dataset: https://rs.figshare.com/collections/ Supplementary_material_from_Coral_reef_fishes_reveal_strong_divergence_in_the_prevalence_of_traits_along_with_the_global_diversity_gradient_/5647995/

All figures have associated raw data.

Field-specific reporting

Please select the one below that is the best fit for your research. If you are not sure, read the appropriate sections before making your selection.

☐ Life sciences ☐ Behavioural & social sciences ☒ Ecological, evolutionary & environmental sciences

For a reference copy of the document with all sections, see nature.com/documents/nr-reporting-summary-flat.pdf

Ecological, evolutionary & environmental sciences study design

All studies must disclose on these points even when the disclosure is negative.

Study description

We investigated the relationships between remoteness and different sources of (local extinction) risk for fish communities. We combined data on reef fish and coral distribution with data on global impacts on oceans. We built networks of potential interactions between fish and corals and between fish and fish (trophic interactions). We used those networks to quantify fish-coral dependency at the global scale. We then combined all the data into a risk assessment framework. We found that while the risks stemming from global hazards from global change and local anthropogenic hazards decrease with remoteness, the risk stemming from ecological dependencies (i.e. risk of cascading effects through interaction networks) increases. This put remote fish communities at risk, obliterating the potential beneficial effect of isolation.

Research sample

The research sample consists of global scale datasets on fish and coral occurrences, fish functional traits, environmental conditions and different impacts on the oceans (e.g. pollution, fisheries, acidification, bleaching risk etc.). We selected ecological traits and environmental layers based primarily on our questions and hypotheses. Then, we chose among available sources based on their completeness. The research sample is meant to represent reef fish communities at the global scale.
We used the following datasets:
1. A global scale reef distribution map (http://data.unep-wcmc.org/datasets/1);
2. The two largest available datasets of species occurrence data, OBIS (https://obis.org/) and GBIF (Actinopterygii, https://doi.org/10.15468/dl.k6vam4; Elasmobranchii, https://doi.org/10.15468/dl.pu4tcx; Holocephali, https://doi.org/10.15468/dl.npckhm; Sarcopterygii, https://doi.org/10.15468/dl.huzujv);
3. The most comprehensive database of species-level information on fish ecology, functional traits and distribution, FishBase (http://www.fishbase.org);
4. A dataset of global maps of ocean impact layers divided into different categories (https://knb.ecoinformatics.org/view/doi:10.5063/F12B8WBS);
5. A friction surface map quantifying the maximum speed at which one individual can travel across a given locality for the whole earth (https://malariaatlas.org/research-project/accessibility-to-cities);
6. A global map of human settlement data (http://data.europa.eu/89h/jrc-ghsl-ghs_smod_pop_globe_r2016a);
7. A dataset including global scale time series of bleaching alert data based on Degree Heating Week (DHW, https://coralreefwatch.noaa.gov);
8. A dataset of global scale climatic and environmental layers (https://www.bio-oracle.org);
9. A global map of marine eco-regions (https://data.unep-wcmc.org/datasets/38);
10. The most complete available dataset on species biotic interactions, GLOBI (https://www.globalbioticinteractions.org);
11. The most complete dataset including data on reef fish abundance in a large set of localities worldwide (Reef Life Survery, https://portal.aodn.org.au/search).
12. An alternative dataset of reef fish distribution (GASPAR) to validate our fish range maps (https://rs.figshare.com/collections/Supplementary_material_from_Coral_reef_fishes_reveal_strong_divergence_in_the_prevalence_of_traits_along_with_the_global_diversity_gradient_/5647995/)

Sampling strategy

We did not predetermine sampling size, and used all available data.

Data collection

Data were downloaded from online sources (using custom Python scripts when possible) by Giovanni Strona using a personal computer and stored both in a physical hard disk and in a remote server owned by the University of Helsinki.

Timing and spatial scale

Data were collected in January 2020. Data cover the global scale.

Data exclusions

From the full list of occurrence records for fish, we excluded all the species for which we could not retrieve ecological information (which resulted in a final list of 5862 fish species). As for the occurrence data, we used a geo-statistical procedure (fully described in methods) to exclude outliers.

Reproducibility	All the data and scripts permitting to replicate the analyses are available from https://github.com/giovannistrona/risk_remoteness.
Randomization	Our study design did not require randomization, as we used all available data in our global analyses. However, we controlled covariates and the potential confounding effect of study effort by performing an extensive set of sensitivity analyses.
Blinding	We used third party data, hence blinding was not relevant for our study.

Did the study involve field work? ☐ Yes ☒ No

Reporting for specific materials, systems and methods

We require information from authors about some types of materials, experimental systems and methods used in many studies. Here, indicate whether each material, system or method listed is relevant to your study. If you are not sure if a list item applies to your research, read the appropriate section before selecting a response.

Materials & experimental systems

n/a	Involved in the study	
☒	☐	Antibodies
☒	☐	Eukaryotic cell lines
☒	☐	Palaeontology and archaeology
☒	☐	Animals and other organisms
☒	☐	Human research participants
☒	☐	Clinical data
☒	☐	Dual use research of concern

Methods

n/a	Involved in the study	
☒	☐	ChIP-seq
☒	☐	Flow cytometry
☒	☐	MRI-based neuroimaging
